# Supplementary material for: LIMA1-alpha staining predicts curative intent surgery response in HPV negative head and neck cancer
Source: EMBO Mol Med. 2025 Jul 17;17(8):2095–114. doi: 10.1038/s44321-025-00266-8 (PMC12340046; doi:10.1038/s44321-025-00266-8)
Supplement: Supplementary file 1 — Appendix [file 44321_2025_266_MOESM1_ESM.pdf]

Appendix

Table of contents	Page number
Appendix Figure S1	2
Appendix Figure S2	3
Appendix Figure S3	4
Appendix Figure S4	5
Appendix Figure S5	6
Appendix Table S1	7
Appendix Table S2	8
Appendix Table S3	8

A

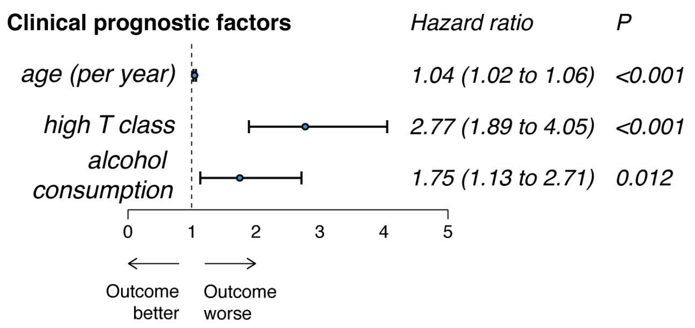

B

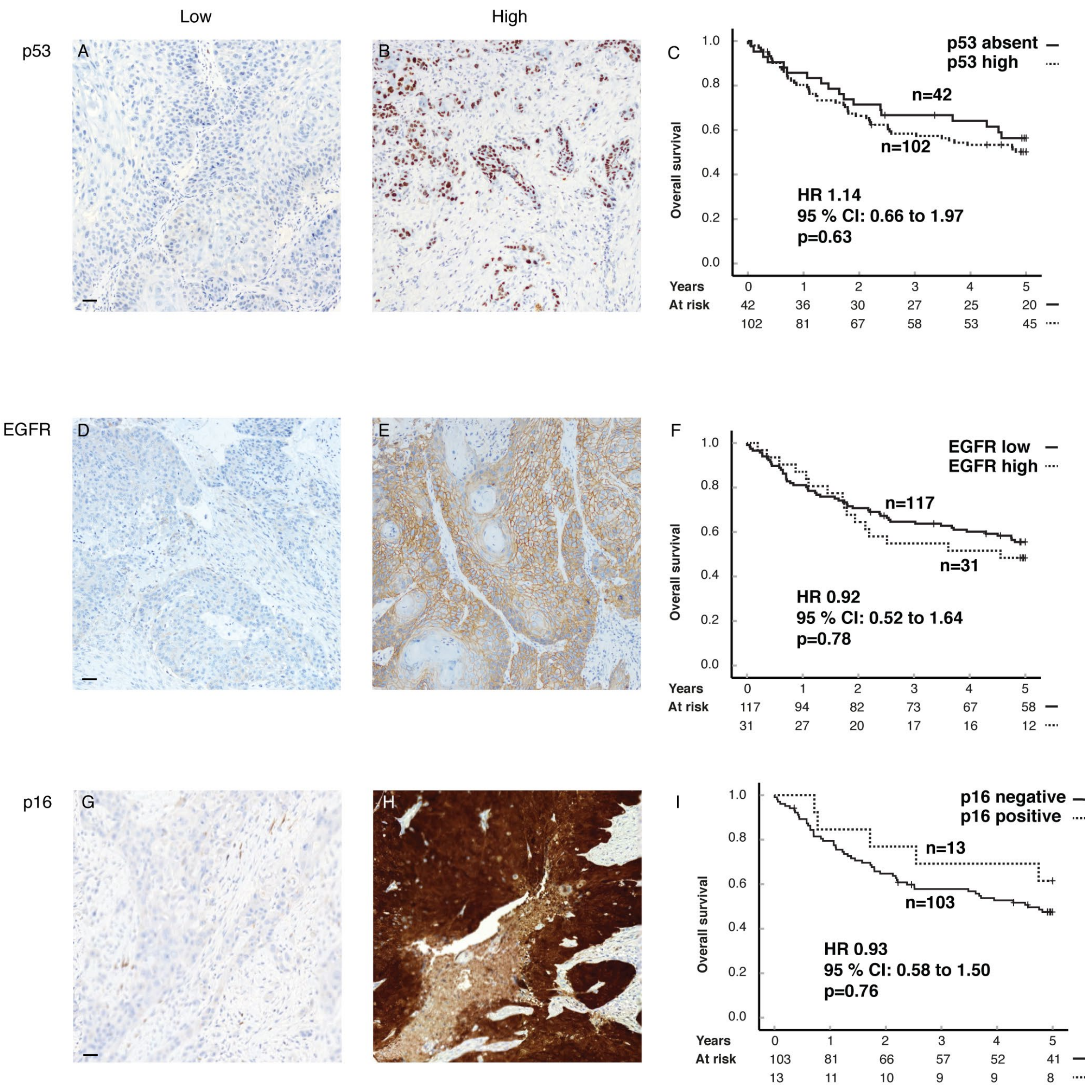

Appendix Figure S1. A) Clinical prognostic factors (age, high T class, and alcohol consumption) used in multivariate modelling (n=312). Multivariable survival analysis was performed constructing Cox propotional hazard models. All scale bars indicated were 100µm. The data is presented by 95% confidence interval. B-G) Representative immunohistochemical stains and prognostic trends (estimates using Kaplan–Meier method and log-rank method for significance) of the investigated biomarkers; p53, EGFR, p16 in HNSCC. Exact p values were as indicated.

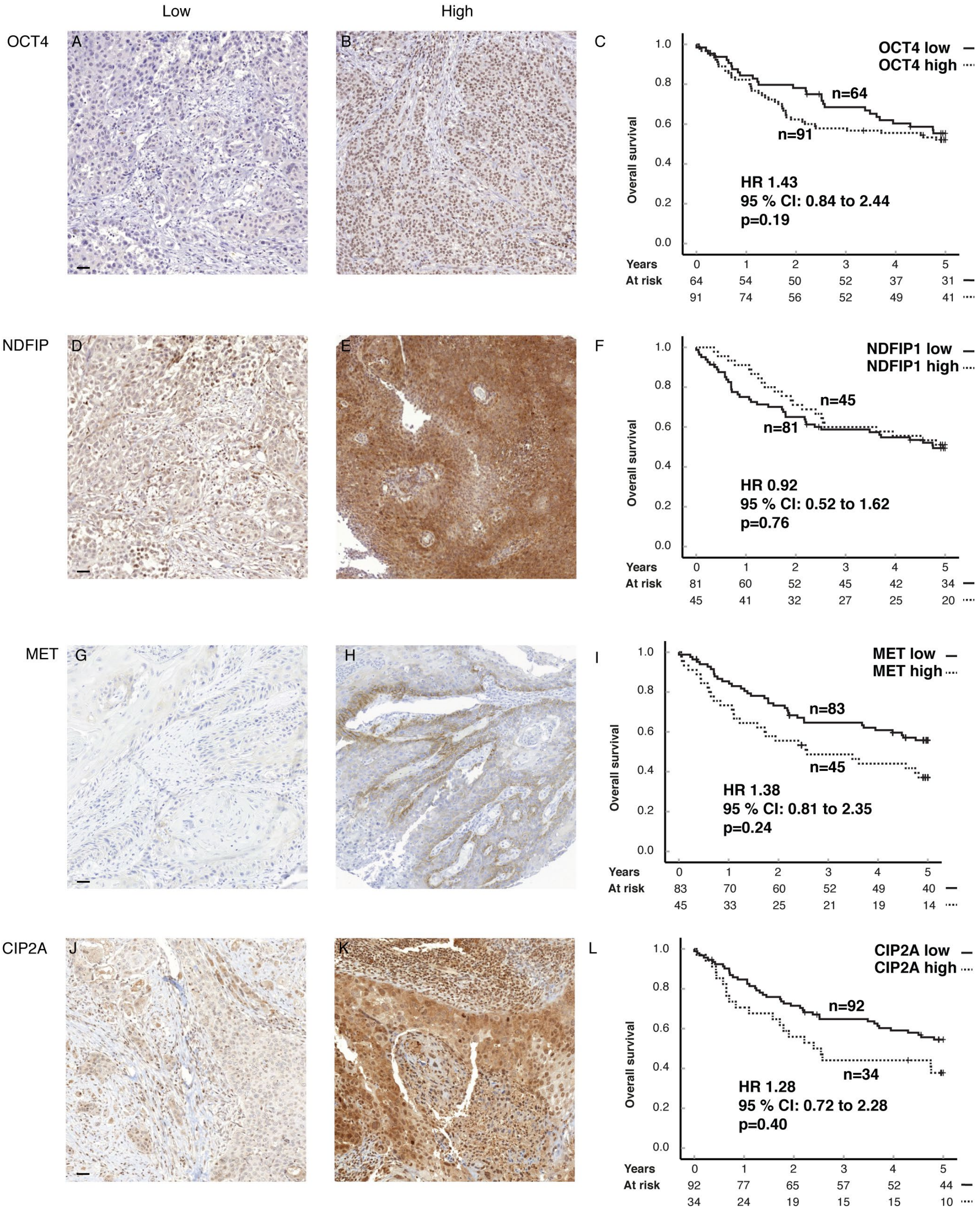

Appendix Figure S2. Representative immunohistochemical stains and prognostic trends (estimates using Kaplan–Meier method and log-rank method for significance) of the investigated biomarkers in HNSCC. A) OCT4, B) NDFIP1, C) MET, D) CIP2A. All scale bars indicated were 100µm.

A

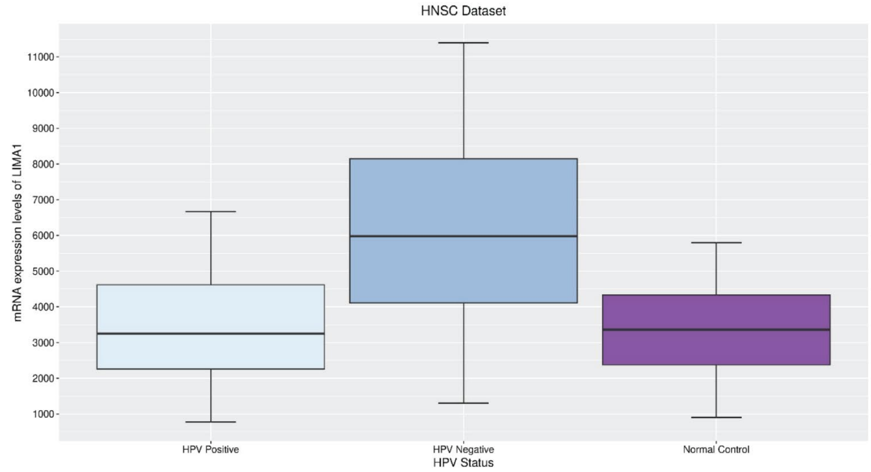

Comparison Group	p-Value	q-Value	Significance
HNSC - HPV(+) vs. HPV(-)	1.15e-1	3.85e-14	YES
HNSC - HPV(+) vs. Normal,	0.93	0.24	NO
HNSC - HPV(-) vs. Normal,	1.91e-10	3.48e-10	YES

B

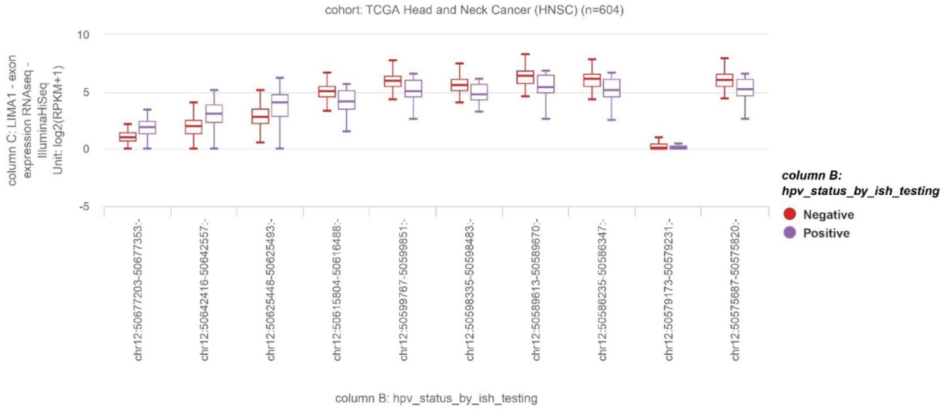

C

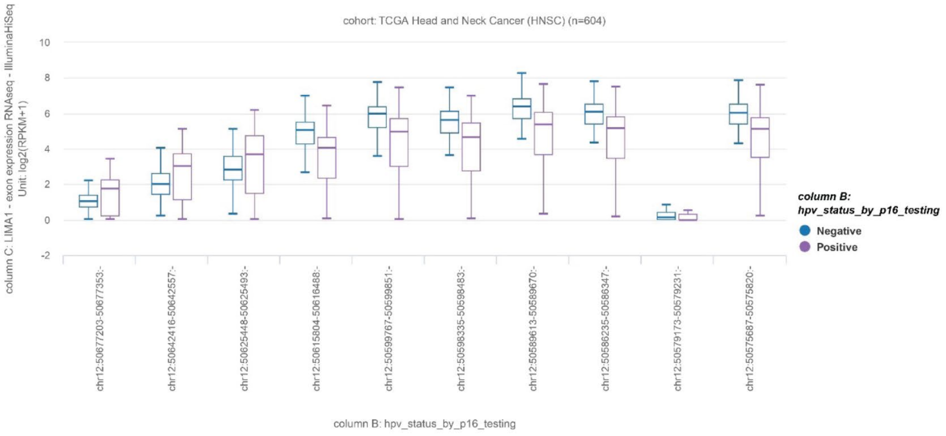

Appendix Figure S3. A) mRNA expression analyses of LIMA1 and HPV in TCGA HNSCC data (n= 554). LIMA1 expression was significantly higher in HPV negative samples in the TCGA data set when compared to HPV positive and normal tissue samples. B-C) Exon level mRNA expression analyses of LIMA1 exons by using

https://xenabrowser.net/. Exons 1-3 (first three exons from the left) are specific for LIMA1-beta isoform and have no significant association to the HPV status when HPV ISH or P16 (n= 604) were analyzed. However, the HPV negative samples had significant correlation with LIMA1 specific exons starting from exon 4.

A

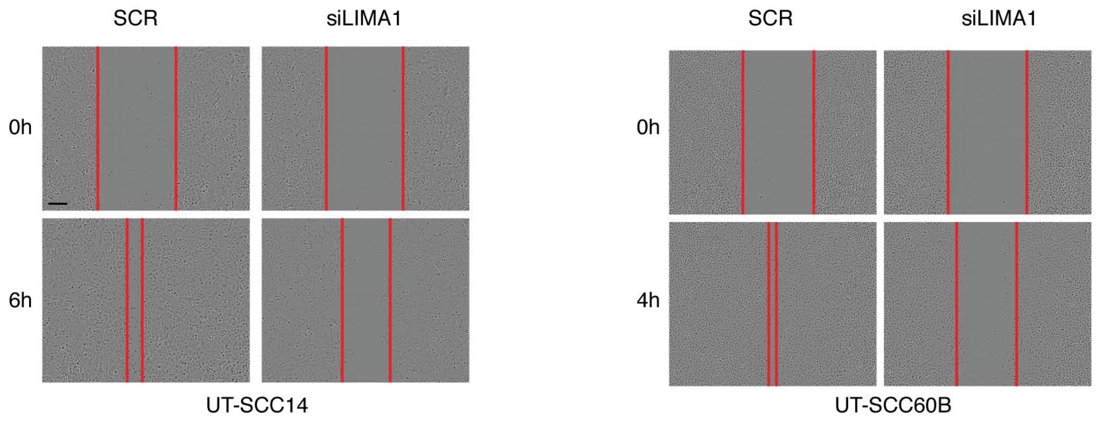

C

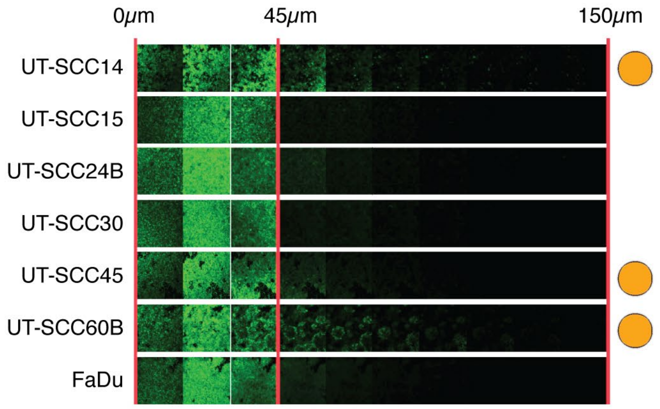

D

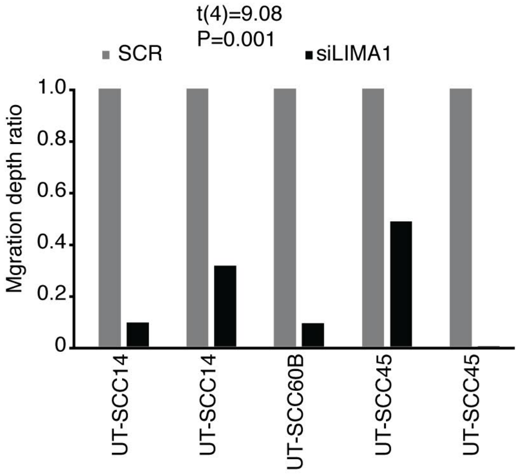

E

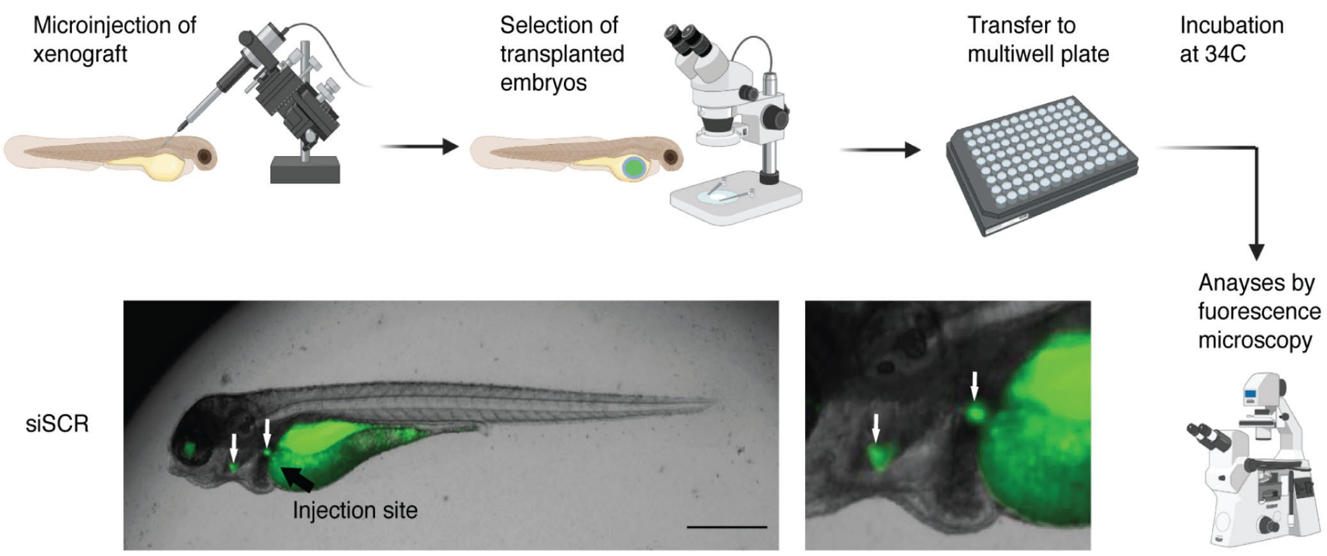

Appendix Figure S4. A-B) In wound healing assay cell migration in UT-SCC-14 and UT-SCC-60B cells decreased after LIMA1 siRNA silencing (Figure EV3). Scale bars indicated were 100μm C) Examination of cell invasive ability in

seven different head and neck cancer cell lines. The distance of migration >45µm is set as cell invasion in invert invasion assay. D) Depletion of LIMA1 using LIMA1-siRNA inhibited cell invasion in UT-SCC-14, UT-SCC-45 and UT-SCC-60B cells as compared to scramble-siRNA (SCR) in five separate experiments. E) Illustration of the workflow of zebrafish embryo xenograft experiment. Fluorescently labeled HNSCC cells were transplanted into zebrafish embryos. Embryos were imaged 4 dpi and tumor size and number of invading cells were quantified.

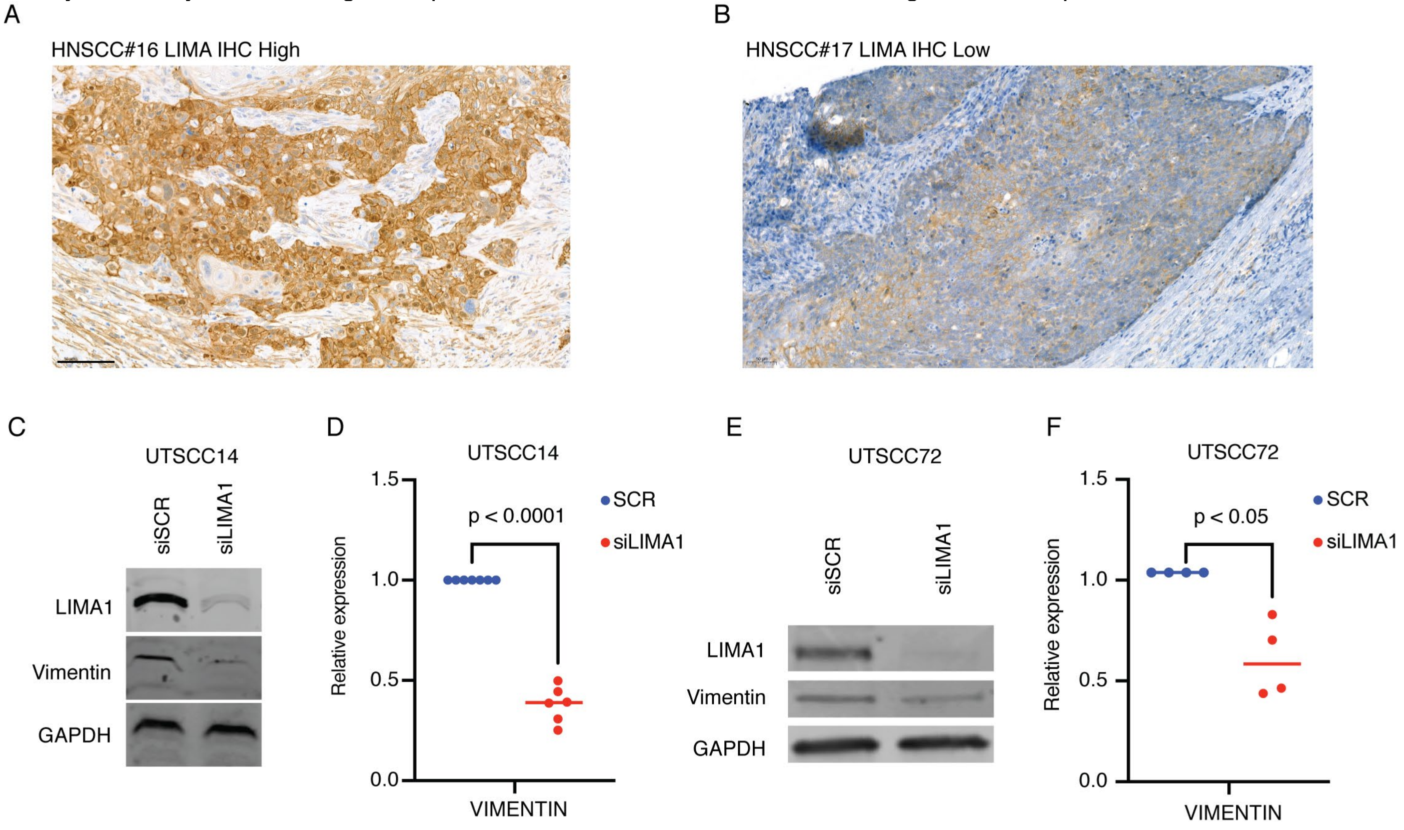

Appendix Figure S5. A-B) Representative LIMA1 IHC stainings of primary HNSCC cancer samples containing High LIMA1 IHC positivity (HNSCC16#) and Low LIMA1 IHC positivity (HNSCC17#). Scale bar indicated were 100µm. C-F) Western blot analysis of vimentin protein levels in two different HNSCC cell lines (UTSCC14 (n=6) and UTSCC72 (n=4)) upon LIMA1 siRNA silencing. The relative expression of vimentin represents vimentin level normalized to loading control GAPDH(n=5). Unpaired two-tailed t-test with Welch's correction was used for statistical analysis. The exact p values were as indicated.

Appendix Table S1. Biomarker staining intensity distribution and multivariable survival analysis (HR, 95 % CI, p value). Cox proportional hazards model controlling for age, primary tumor site, T class, and alcohol use.

		**T any N0/PV-TMA**		**Survival effect**	
		n	*%*	*HR*	*p*
**p53**					
	absent	42	29 %	1	-
	wt or high	102	71 %	1.14 (0.66 to 1.97)	0.63
**EGFR**					
	low-moderate	117	79 %	1	-
	strong	31	21 %	0.92 (0.52 to 1.64)	0.78
**CIP2A**					
	low-moderate	92	73 %	1	-
	high	34	27 %	1.28 (0.72 to 2.28)	0.40
**Oct4**					
	negative	64	41 %	1	-
	positive	91	59 %	1.43 (0.84 to 2.44)	0.19
**p16**					
	negative	103	89 %	1	-
	positive	13	11 %	0.93 (0.58 to 1.50)	0.76
**NDFIP1**					
	negative	81	64 %	1	-
	positive	45	36 %	0.92 (0.52 to 1.62)	0.76
**MET**					
	low	83	65 %	1	-
	moderate-high	45	35 %	1.38 (0.81 to 2.35)	0.24
**LIMA1**					
	low	63	50 %	1	-
	moderate-high	63	50 %	2.10 (1.24 to 3.58)	0.006

Appendix Table S2. Characteristics of HNSCC patients selected for the prospective follow-up study. RT (post operative radiotherapy), CRT (post operative chemoradiotherapy).

HNSCC ID	HNSCC origing	Gender	Age	T	N	M
#1	Mandibule	Male	62	2	0	0
#2	Tongue	Female	55	3	0	0
#3	Mandibule	Female	63	2	0	0
#4	Cheek	Male	70	2	0	0
#5	Maxillary sinus	Male	73	4	0	0
#6	Mandibule	Female	88	4	0	0
#7	Tongue	Male	58	3	1	0
#8	Tongue	Male	44	3	2	0
#9	Base of mouth	Male	51	3	2	0
#10	Tongue	Female	96	3	0	0
#11	Tongue	Male	65	2	1	0
#12	Mandibule	Female	88	4	1	0
#13	Tongue	Female	70	2	2	0
#14	Tongue	Female	54	3	0	0
#15	Tongue	Female	80	2	0	0

Appendix Table S3. Summary of p16 and LIMA1 IHC analysis results for HNSCC patients in Cohorts 1, 3, 4 and 5

	p16 positive		p16 negative		p16 data missing		n
	n	%	n	%	n	%	
Cohort 1	13	10.2	103	80.4	12	9.4	128
Cohort 3	0	-	7	46.7	8	53.3	15
Cohort 4	8	4.3	175	94.6	2	1.1	185
Cohort 5	4	4.2	38	40	53	55.8	95
**In total**	**25**	**5.9**	**323**	**76.3**	**75**	**17.7**	**423**

	LIMA1 Low		LIMA1 High		LIMA1 data missing		n
	n	%	n	%	n	%	
Cohort 1	63	49.2	63	49.2	2	1.6	128
Cohort 3	5	33.3	10	66.7	0	0	15
Cohort 4	116	62.7	68	36.8	1	0.5	185
Cohort 5	44	46.3	42	44.2	9	9.5	95
**In total**	**228**	**53.9**	**183**	**43.3**	**12**	**2.8**	**423**
