# Supplementary figures and images for: LIMA1-alpha staining predicts curative intent surgery response in HPV negative head and neck cancer
Source: EMBO Mol Med. 2025 Jul 17;17(8):2095–114. doi: 10.1038/s44321-025-00266-8 (PMC12340046; doi:10.1038/s44321-025-00266-8)

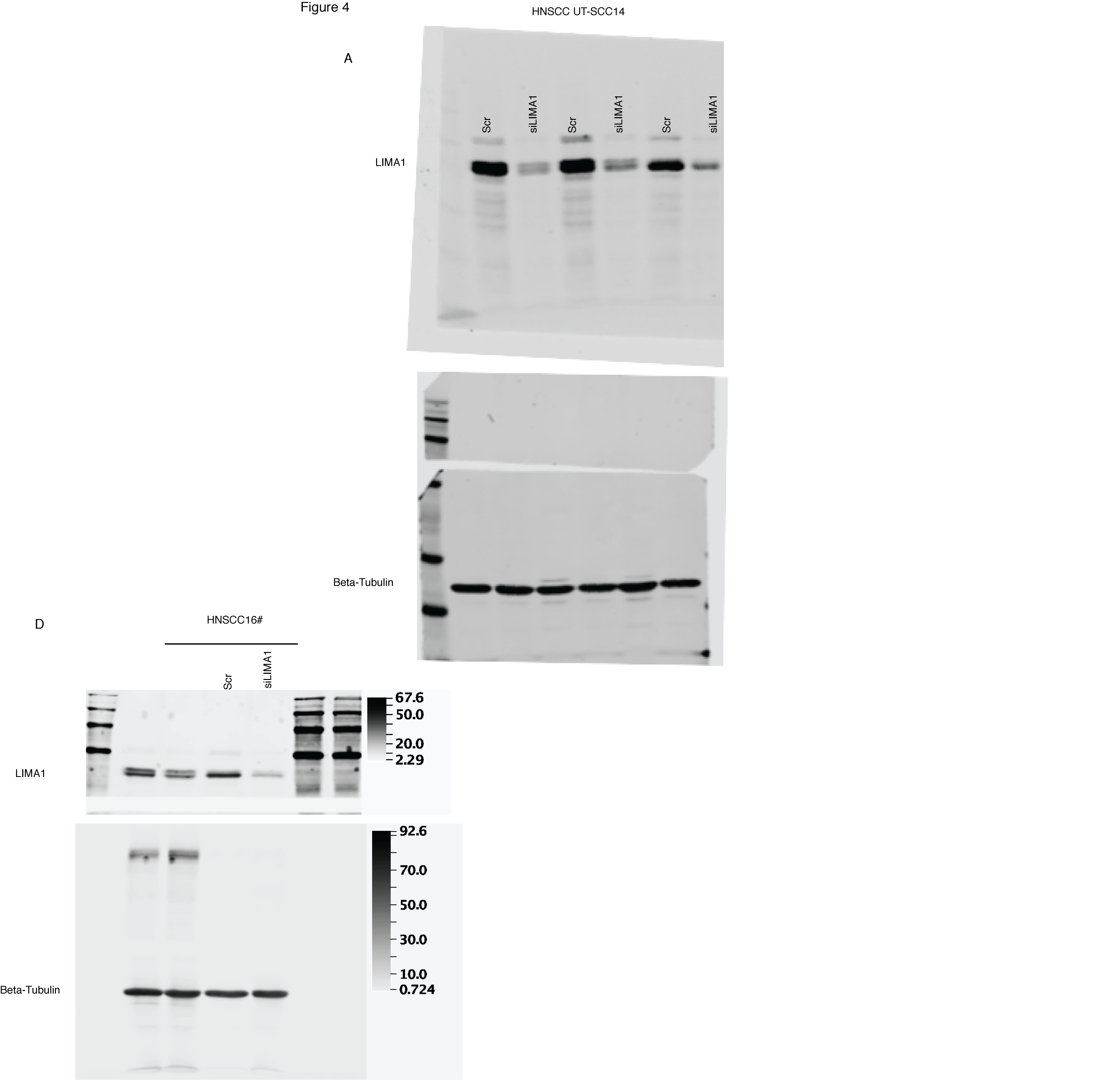

Supplement: Supplementary file 4 — Source data Fig. 4 [file 44321_2025_266_MOESM4_ESM.zip › Figure4 2/Figure 4 A and D original western blots.png]

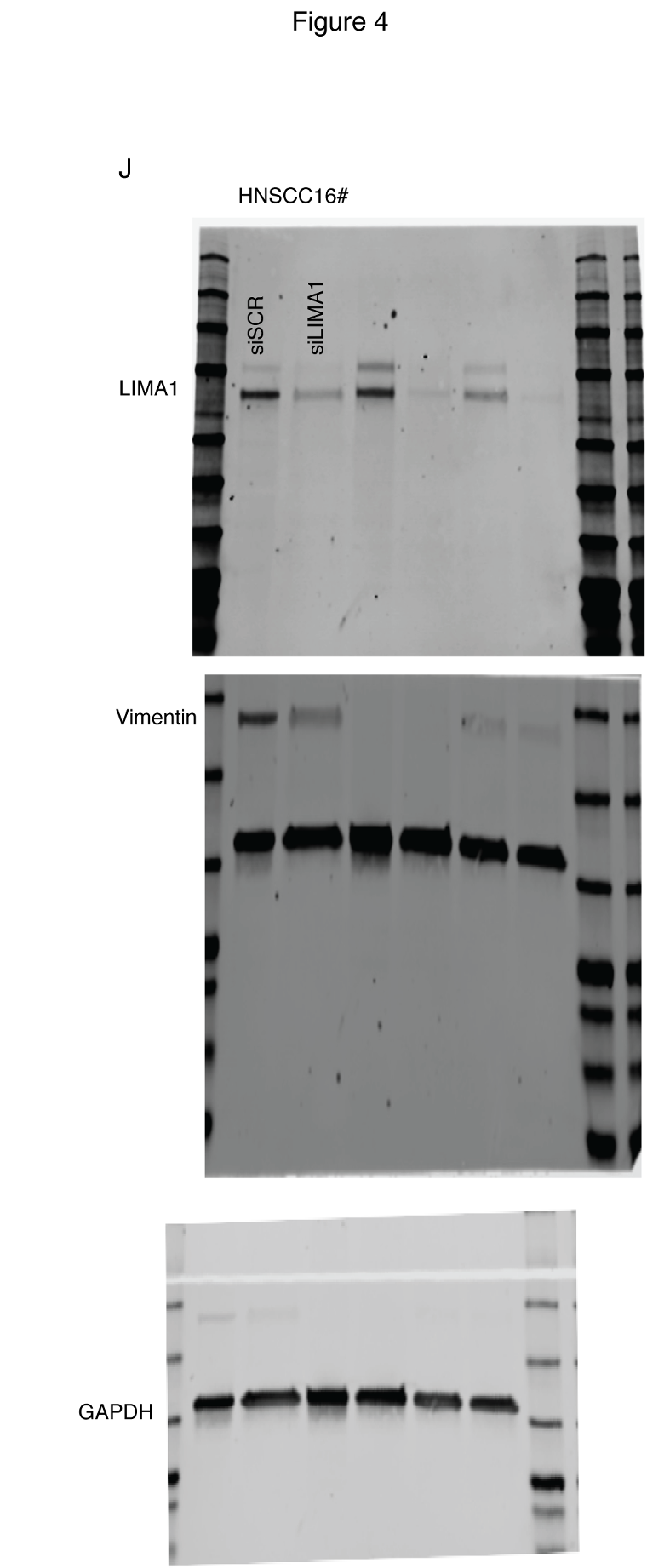

Supplement: Supplementary file 4 — Source data Fig. 4 [file 44321_2025_266_MOESM4_ESM.zip › Figure4 2/Figure 4 J original western blots.png]

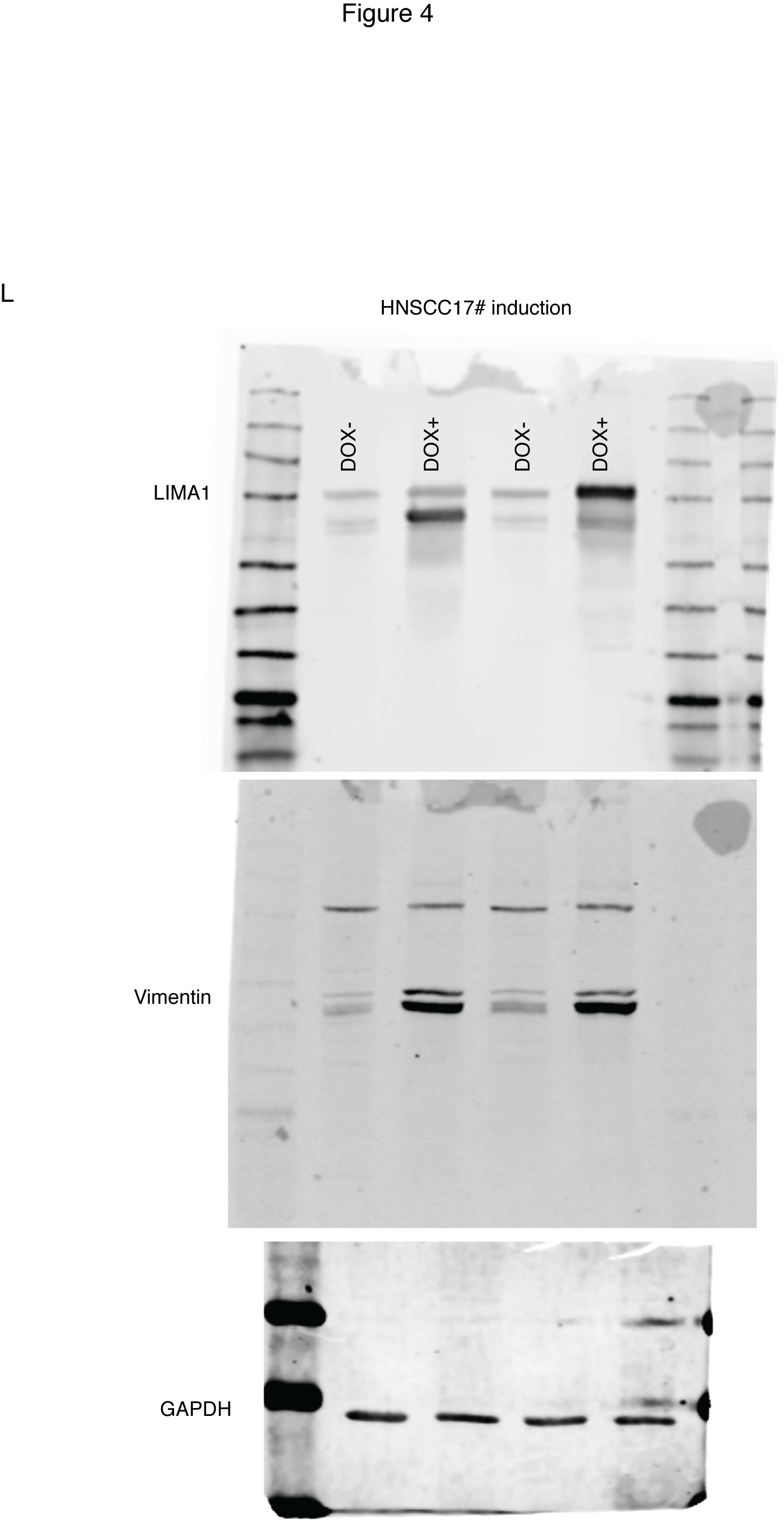

Supplement: Supplementary file 4 — Source data Fig. 4 [file 44321_2025_266_MOESM4_ESM.zip › Figure4 2/Figure 4 L original western blots.png]

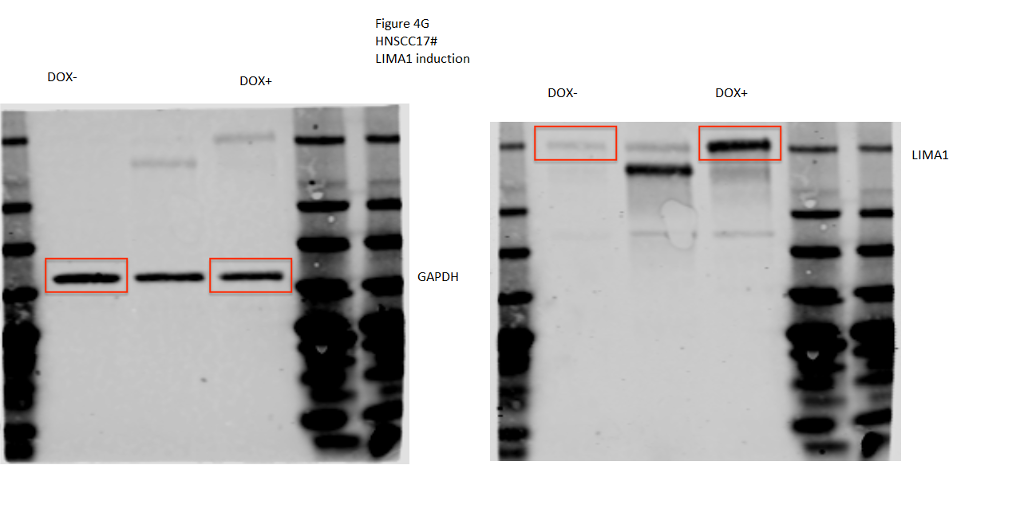

Supplement: Supplementary file 4 — Source data Fig. 4 [file 44321_2025_266_MOESM4_ESM.zip › Figure4 2/Figure 4 G original western blots.png]
