# Supplementary material for: LIMA1-alpha staining predicts curative intent surgery response in HPV negative head and neck cancer
Source: EMBO Mol Med. 2025 Jul 17;17(8):2095–114. doi: 10.1038/s44321-025-00266-8 (PMC12340046; doi:10.1038/s44321-025-00266-8)
Supplement: Supplementary file 5 — Expanded View Figures [file 44321_2025_266_MOESM5_ESM.pdf]

Expanded View Figures

Figure EV1. Validation of LIMA1 antibodies.

(**A**) Schematic presentation of LIMA1-alpha and -beta isoforms and the antigen regions used for raising the LIMA1 antibodies used in this study. (**B**) Western blot characterization of LIMA1 expression (HPA023871 antibody) in 14 different HNSCC cancer cell lines and (**C**) in 13 triple-negative breast cancer cell lines. (**D**) Western full blot analysis of LIMA1 antibody (SC-136399) specificity after LIMA1 silencing with six different LIMA1 siRNA transfections. (**E**) Western full blot characterization of custom-made polyclonal LIMA1-beta specific antibody (RB581). UT-SCC-14 and -72 are highly LIMA1-alfa-positive HNSCC cell lines. HN09 HNSCC cancer cell line containing low LIMA1-beta expression were transduced either with LIMA1-alpha (a-induced) or LIMA1-beta (b-induced) lentiviral Tet-inducible gene expression vector. LIMA1 expression and antibody specificity upon Doxycycline treatment were ensured by western blot with LIMA1-beta specific antibody (RB581). (**F**, **G**) Western blot and immunofluorescence analyzes of HPA023871 and SC-136399 antibodies after LIMA1 siRNA silencing of patient-derived HNSCC cell lines. (**H**) Specificity in immunofluorescence of LIMA1-beta specific antibody (RB581) was ensured by conducting RB581 IF staining in HNSCC cell lines containing both LIMA1-alpha and -beta expression (MISB10) and with the cell line lacking LIMA1-beta expression (UT-SCC-14). Scale bars indicated were 10 µm.

© The Author(s)

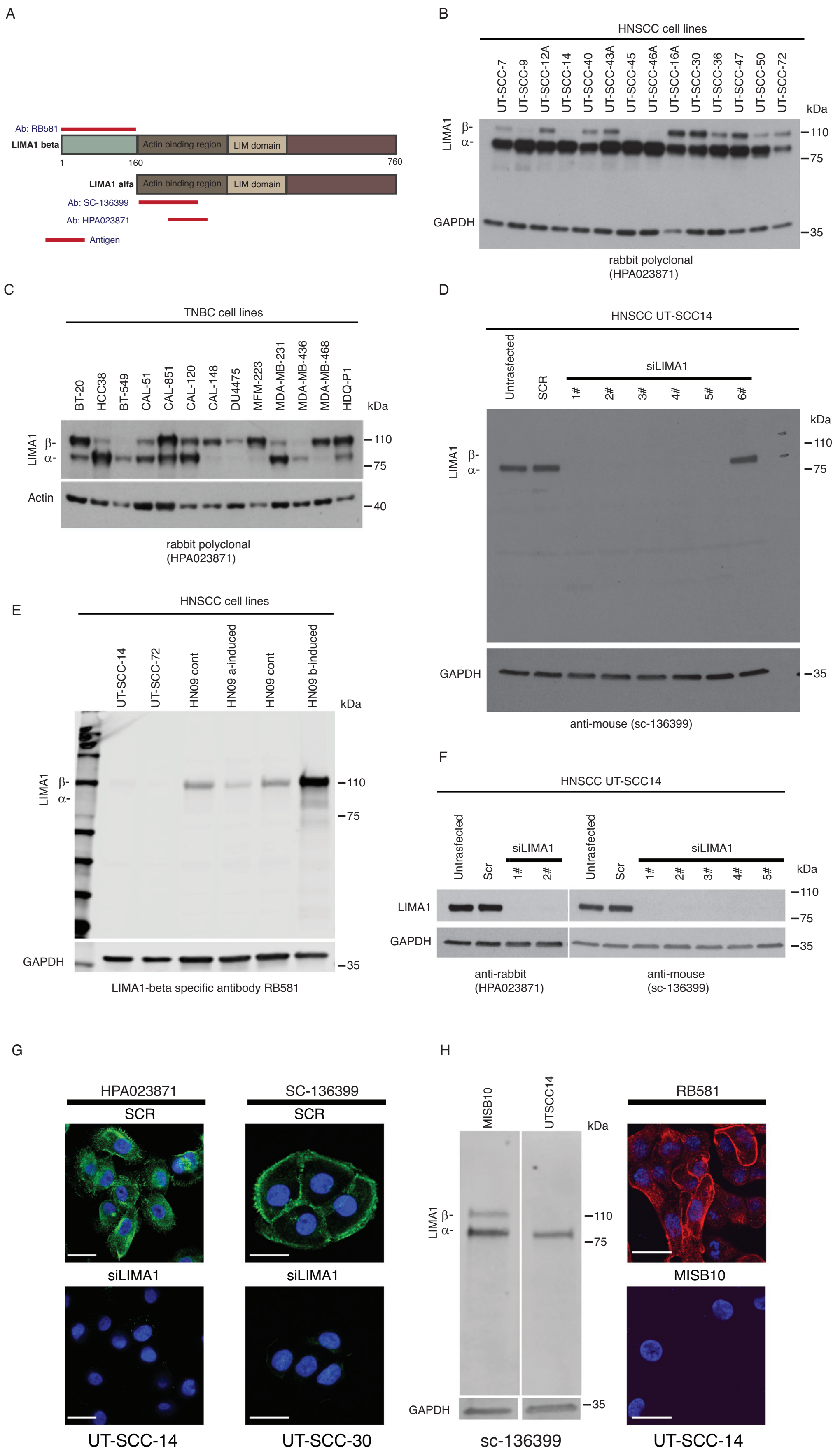
A
Ab: RB581
LIMA1 beta
Actin binding region
LIM domain
1
160
760
LIMA1 alfa
Actin binding region
LIM domain
Ab: SC-136399
Ab: HPA023871
Antigen
B
HNSCC cell lines
UT-SCC-7
UT-SCC-9
UT-SCC-12A
UT-SCC-14
UT-SCC-40
UT-SCC-43A
UT-SCC-45
UT-SCC-46A
UT-SCC-16A
UT-SCC-30
UT-SCC-36
UT-SCC-47
UT-SCC-50
UT-SCC-72
kDa
LIMA1
β-
α-
110
75
GAPDH
35
rabbit polyclonal
(HPA023871)
C
TNBC cell lines
BT-20
HCC38
BT-549
CAL-51
CAL-851
CAL-120
CAL-148
DU4475
MFM-223
MDA-MB-231
MDA-MB-436
MDA-MB-468
HDQ-P1
kDa
LIMA1
β-
α-
110
75
Actin
40
rabbit polyclonal
(HPA023871)
D
HNSCC UT-SCC14
Untrasfected
SCR
siLIMA1
1#
2#
3#
4#
5#
6#
kDa
110
75
LIMA1
β-
α-
GAPDH
35
anti-mouse (sc-136399)
E
HNSCC cell lines
UT-SCC-14
UT-SCC-72
HN09 cont
HN09 a-induced
HN09 cont
HN09 b-induced
kDa
LIMA1
β-
α-
110
75
GAPDH
35
LIMA1-beta specific antibody RB581
F
HNSCC UT-SCC14
Untrasfected
Scr
siLIMA1
1#
2#
Untrasfected
Scr
siLIMA1
1#
2#
3#
4#
5#
kDa
LIMA1
110
75
GAPDH
35
anti-rabbit
(HPA023871)
anti-mouse
(sc-136399)
G
HPA023871
SCR
siLIMA1
SC-136399
SCR
siLIMA1
UT-SCC-14
UT-SCC-30
H
MISB10
UTSCC14
kDa
LIMA1
β-
α-
110
75
GAPDH
35
sc-136399
RB581
MISB10
UT-SCC-14

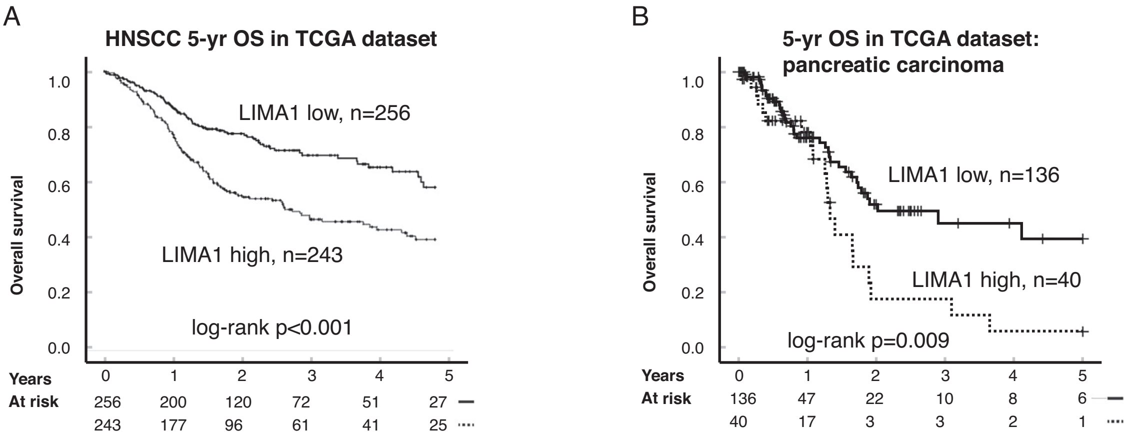

Figure EV2. Prognostic role for LIMA1 in TCGA HNSCC and pancreatic cancer data.

(**A**, **B**) The prognostic effect of LIMA1 was confirmed in TCGA HNSCC ($P < 0.001$) and operatively treated pancreatic cancer ($P = 0.009$) datasets.

 © The Author(s)

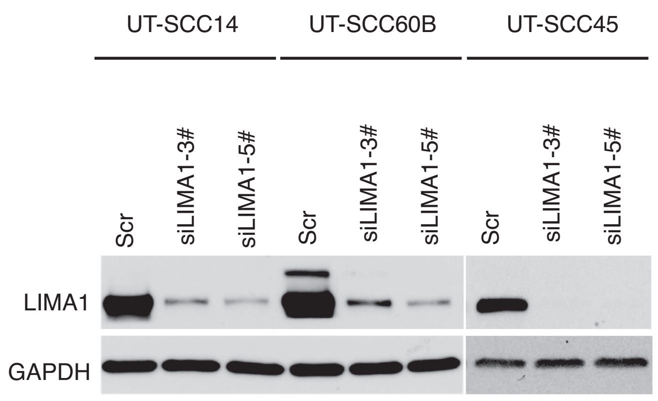

Figure EV3. Validation of LIMA1 protein inhibition by siRNA.

Depletion of LIMA1 in three different patient-derived HNSCC cell lines (UT-SCC14, UT-SCC60B and UT-SCC45).

© The Author(s)

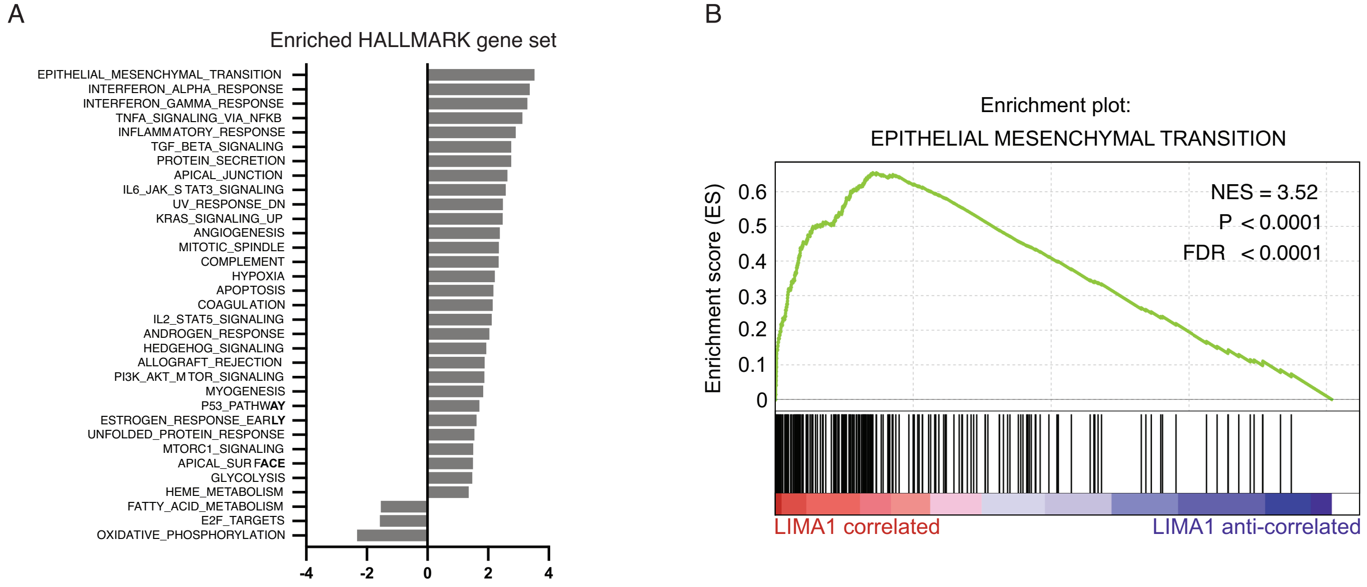

Figure EV4. LIMA1 promotes epithelial mesenchymal transition.

(**A**) Gene set enrichment analysis (GSEA) for genes co-expressing with LIMA1 in TCGA HNSCC data set. The normalized enrichment scores (NES) for HALLMARK gene sets with FDR < 0.05 are shown. (**B**) Enrichment plot for HALLMARK EMT gene set ($P < 0.0001$) from GSEA analysis. Default statistics of GSEA was used (Subramanian et al, 2005).

 © The Author(s)
